# Effects of a resistance training intervention on the strength-deficit of elite young soccer players

**DOI:** 10.5114/biolsport.2022.106157

**Published:** 2021-07-28

**Authors:** Irineu Loturco, Lucas A. Pereira, Chris Bishop, Vinicius Zanetti, Tomás T. Freitas, Fernando Pareja-Blanco

**Affiliations:** 1 NAR – Nucleus of High Performance in Sport, São Paulo, Brazil; 2 Department of Human Movement Sciences, Federal University of São Paulo, São Paulo, Brazil; 3 University of South Wales, Pontypridd, Wales, United Kingdom; 4 Faculty of Science and Technology, London Sports Institute, Middlesex University, London, UK; 5 Red Bull Brazil Football, Jarinú, Brazil; 6 Faculty of Sports Sciences, Catholic University of Murcia, Murcia, Spain; 7 UCAM Research Center for High Performance Sport – Catholic University of Murcia, UCAM, Spain; 8 Physical Performance and Sports Research Center, Pablo de Olavide University, Seville, Spain; 9 Faculty of Sports Sciences, Department of Sports and Computers Sciences, Pablo de Olavide University, Seville, Spain

**Keywords:** Team-sports, Athletic performance, Muscle power, Maximum dynamic strength, Elite athletes

## Abstract

The aim of this study was to examine the effects of a traditional resistance training scheme on the relative strength (RS), relative peak-force (RPF), strength deficit (SDef), and vertical jump and sprint abilities in elite young soccer players. Thirty-five under-20 soccer players from two professional clubs were assessed before and after a 4-week competitive period. One team performed 12 sessions of a structured resistance training program and the other maintained their regular soccer-specific training and competitive routines. Resistance training sessions consisted of half-squat and jump-squat exercises at intensities of 30–80% of the one-repetition maximum. Both teams performed pre- and post-measurements in the following order: (1) countermovement jump (CMJ), (2) 20-m sprint, and (3) half-squat one-repetition maximum to determine the RS, RPF, and SDef. A two-way analysis of variance was used to test for group x time interaction among variables. Effect sizes (ES) and 95% confidence intervals (CI) were also calculated. Group x time interactions were demonstrated for RS ([ES [95%CI] = 1.21 [0.57; 1.85], *P* = 0.001), RPF (ES [95%CI] = 1.18 [0.52; 1.80], *P* = 0.001), SDef (ES [95%CI] = 0.86 [0.01; 1.71], *P* = 0.04), and CMJ (ES [95%CI] = 0.64 [0.28; 0.99], *P* = 0.001); whereas a non-significant interaction was observed for 20-m sprint performance (ES [95%CI] = 0.02 [-0.32; 0.36], *P* = 0.85). Traditional strength-power oriented training resulted in improved maximum strength performance and CMJ ability but, paradoxically, increased the SDef. As a consequence, stronger athletes are not necessarily able to use greater percentages of their peak-force against relatively lighter loads.

## INTRODUCTION

Muscle strength is considered one of the most important attributes among a set of qualities that have a significant impact on sport performance [1]. Furthermore, the ability to apply high levels of force at high velocities (i.e., power) is also recognized for its crucial role in many sport tasks, such as sprinting and jumping [2, 3]. Although maximum strength and power have been extensively investigated and described in the literature [1, 2], there is still a need to better understand the individual capacity to exert large amounts of force when submaximal loads are lifted [4].

The strength-deficit (SDef) represents the relative difference between the force produced against the “one-repetition maximum” (i.e., 1RM) and any other load of lower magnitude (e.g., 40–50% 1RM) [1, 5]. In practical terms, a reduction in the SDef suggests that an athlete is applying higher relative force against a given %1RM [1, 5]. Conversely, an increased SDef indicates that an athlete may not be able to exploit his/her maximum strength capacity during unloaded (and faster) sport tasks [1, 4]. A recent study comparing sprinters and rugby players revealed that sprinters present higher levels of relative strength (RS) and relative peak-force (RPF) (i.e., 1RM and peak-force values normalized to body-mass), and lower levels of SDef, being able to produce greater amounts of force (relative to their maximum dynamic force) at the low-force/high-velocity portion of the force-velocity relationship [4]. Although no differences in maximum strength and absolute peak-force were found between these groups, the superior capacity of sprinters to apply force against lighter loads and at higher velocities may also be connected to their superior sprint and jump performance [4]. Therefore, it can be assumed that, in sports where sprint and jump abilities are decisive factors (e.g., soccer) [6, 7], resistance training programs should be designed not only to increase the strength level, but also to reduce the SDef.

Despite the importance of SDef, to date, no studies have examined the effects of a structured training intervention on this strength-derived parameter. The current study analyzed the effects of a resistance training program on the RS, RPF, and SDef of under-20 soccer players. Additionally, we assessed the effects of this training scheme on sprint and jump capacities.

## MATERIALS AND METHODS

### Participants

Thirty-five under-20 Brazilian soccer players (18.4 ± 0.7 years; 178.1 ± 6.0 cm; 71.8 ± 6.4 kg) from two professional clubs participated in this study. Both teams were assessed during the inseason phase, after an inter-season preparatory period of ~ 4 weeks and participated in similar tournament divisions. At the time of the study, soccer players had at least 5 years of experience in a professional soccer academy, being frequently exposed to different and complementary strength and conditioning practices. The research was approved by the local Ethics Committee and athletes signed an informed consent prior to participation.

### Study Design

This quasi-experimental longitudinal study assessed the effects of a structured strength-training intervention on distinct strength parameters of elite young soccer players. Athletes were assessed before and after a 4-week in-season period. One team (experimental group; n = 18) performed 12 sessions of a traditional resistance training program (Table 1) [8]. Basically, the resistance training sessions consisted of performing half-squats or jump-squats at fixed percentages of 1RM and moving the bar as fast and as powerfully as possible during the concentric portion of the lift. In addition, the experimental group played, on average, one official and/or one friendly/simulated match per week. The other team (control group; n = 17) was competing in two simultaneous tournaments, being involved in 2 official matches per week in different regions of the country, thus precluding the implementation of a regular resistance training program. Both teams completed the pre- and post-assessments on the same day, in the following order: countermovement jump (CMJ), 20-m sprint, and a progressive loading test up to the 1RM. All players were well familiarized with the testing procedures due to their regular testing routines in our high-performance training center. Subjects were required to be in a fasting state for at least 2-h, avoiding caffeine and alcohol consumption for 24-h before the procedures. Prior to the test, the athletes performed a standardized warm-up protocol including general (i.e., running at a moderate pace for 10-min followed by dynamic lower limb stretching for 3-min) and specific exercises (i.e., submaximal attempts of each test).

**TABLE 1:** Training scheme for the traditional strength training group over the 4-week period.

2 Sessions	2 sessions	2 sessions	6 sessions
*Half-Squat*	*Half-Squat*	*Half-Squat*	*Jump-Squat*
6 sets of 10 repetitions	6 sets of 8 repetitions	6 sets of 6 repetitions	6 sets of 4–6 repetitions
60% 1RM	70% 1RM	80% 1RM	30 % 1RM

1 RM: one-repetition maximum.

### Procedures

#### Countermovement Jump

To perform the CMJ, players were required to execute a downward movement followed by complete extension of the legs, freely determining the countermovement amplitude to avoid changes in jumping coordination. All jumps were performed on a contact platform (Elite Jump®, S2 Sports, São Paulo, Brazil) with the hands on the hips. Five attempts were allowed, interspersed by 15-s intervals. The highest jump was recorded.

#### Sprinting speed

Two pairs of photocells (Smart-Speed, Fusion-Sport, Brisbane, Australia) were positioned at the starting line and at a distance of 20-m on an indoor track. Athletes sprinted twice, starting from a standing position, 0.5-m behind the starting line. A 5-min rest interval was allowed between attempts and the best trial was recorded.

#### Progressive loading test in the half-squat exercise

Maximum strength was assessed using the half-squat 1RM test, as described previously [4, 9, 10]. Prior to the test, athletes executed a warm-up set, which consisted of 5 repetitions between 40 and 60% of the estimated 1RM. Three minutes after the warm-up, athletes were allowed up to 5 attempts at ~70, 80, 90, and > 95% of the estimated 1RM to obtain the actual 1RM value [9, 10]. A 3-min rest interval was provided between all repetitions [9]. The test was performed on a Smith-machine device (Hammer-Strength Equipment, Rosemont, USA). Athletes were instructed to move the barbell as fast as possible during the concentric phase of movement in all attempts. The PF was continuously assessed during all attempts at a sampling rate of 1,000 Hz by a linear velocity transducer (T-Force Dynamic Measurement System; Ergotech Consulting S.L., Murcia, Spain) attached to the Smith-machine barbell. SDef was calculated as the percentage difference between peak-force at the lighter load (i.e., 40% 1RM) and at 1RM. The 1RM and PF at 1RM values were normalized to the body-mass (i.e., RS and RPF) for analysis purposes.

### Statistical Analyses

The statistical analyses were performed with the JASP software package version 0.13 for Windows (Department of Psychological Methods, University of Amsterdam, Amsterdam, The Netherlands). Data normality was checked via the Shapiro-Wilk test. A two-way analysis of variance with repeated measures was used to analyze between and within group differences as well as group x time interactions among variables. The Bonferroni post-hoc test identified where significant differences occurred. The magnitudes of the differences were expressed as effect sizes (ES) [11] along with their 95% confidence intervals (CI). The magnitudes of the ES were interpreted using the following thresholds: < 0.2, 0.2–0.6, 0.6–1.2, 1.2–2.0, 2.0–4.0, and > 4.0 for trivial, small, moderate, large, very large, and near perfect, respectively [12]. Significance level was set at *P* < 0.05. All tests used here presented small errors of measurement, as evidenced by their high levels of accuracy and reproducibility (i.e., coefficient of variation < 10% and intraclass correlation coefficient > 0.90 for all assessments) [12].

## RESULTS

**FIG. 1:**
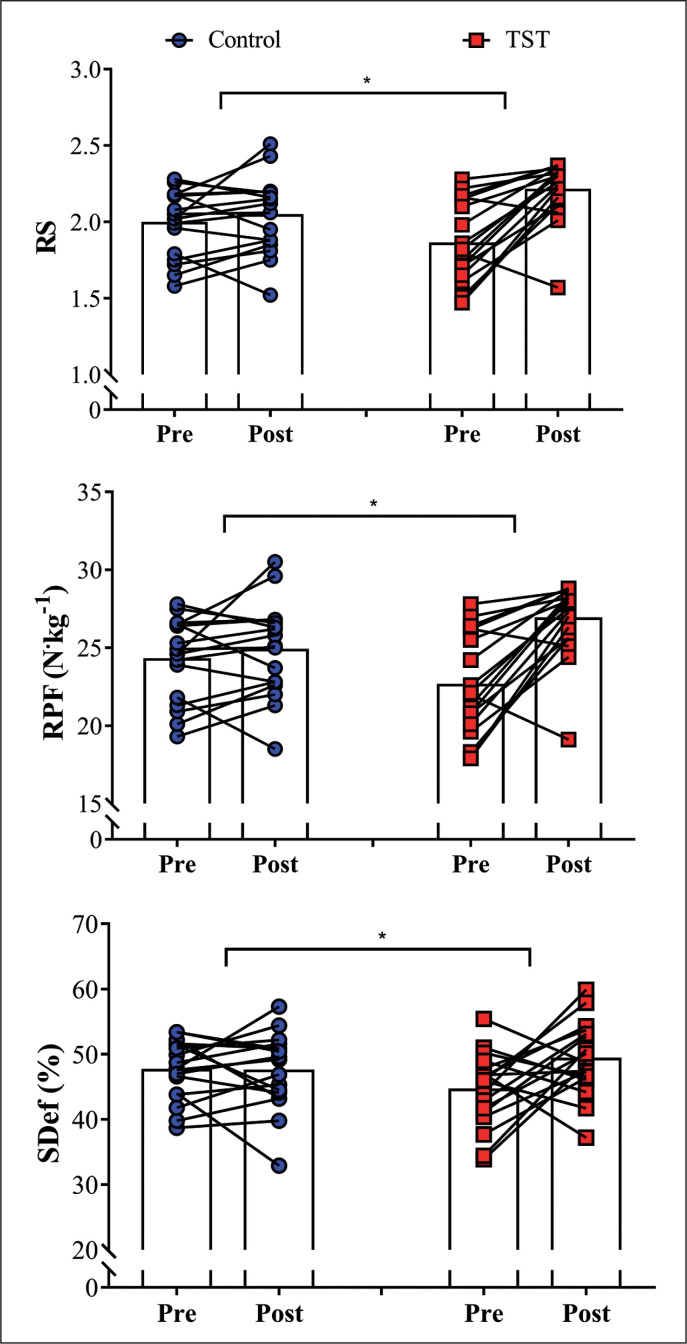
Comparisons between pre- and post-measures in both groups for relative strength (RS), relative peak-force (RPF), and strength deficit (SDef) assessed in the half-squat exercise. Control: control group (n = 17); TST: traditional strength training (n = 18). ^#^TST group showed a significant increase in all strength-derived parameters, when comparing pre- and post-measures (*P* < 0.05). *indicates significant group x time interaction, *P* < 0.05.

No between group differences were observed in the baseline measures for any variables (*P* = 0.12, 0.11, 0.09, 0.44, for RS, RPF, SDef, and CMJ, respectively), with the exception of sprint performance (*P* = 0.001). Figure 1 depicts the comparisons between pre- and post-measures in both groups for RS, RPF, and SDef. A main effect of time was observed in the TST group for RS (ES [95%CI] = 1.25 [0.76; 1.73], *P* < 0.001), RPF (ES [95%CI] = 1.20 [0.75; 1.65], *P* < 0.001), and SDef (ES [995%CI] = 0.79 [0.08; 1.51], *P* = 0.03). In contrast, no significant differences in pre- vs post-test comparisons were observed for the control group for RS (ES [95%CI] = 0.22 [-0.19; 0.63], *P* = 0.27), RPF (ES [95%CI] = 0.24 [-0.13; 0.61], *P* = 0.28), and SDef (ES [995%CI] = -0.03 [-0.59; 0.53], *P* = 0.92). Group x time interactions were demonstrated for all variables (RS: ES [95%CI] = 1.21 [0.57; 1.85], *P* = 0.001; RPF: ES [95%CI] = 1.18 [0.52; 1.80], *P* = 0.001; SDef: ES [95%CI] = 0.86 [0.01; 1.71], *P* = 0.04).

**FIG. 2:**
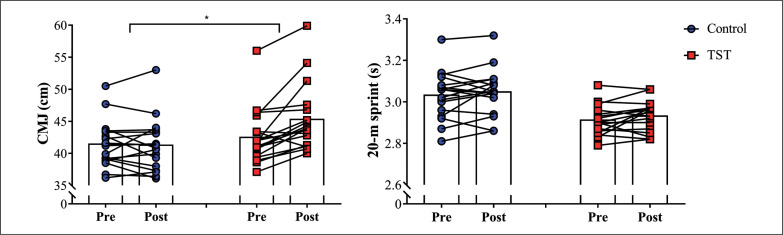
Comparisons between pre- and post-measures in both groups for countermovement jump (CMJ) height and 20-m sprint time. Control: control group (n = 17); TST: traditional strength training (n = 18). ^#^TST group showed a significant increase in CMJ height, (*P* < 0.05) when comparing pre- and post-measures. *indicates significant group x time interaction, *P* < 0.05.

Figure 2 shows the comparisons between pre- and post-measures in both groups for CMJ and 20-m sprint. A main effect of time was observed in the TST group for CMJ (ES [95%CI] = 0.63 [0.32; 0.93], *P* < 0.001), but not for 20-m sprint performance (ES [95%CI] = 0.25 [-0.10; 0.61], *P* = 0.13). In addition, no significant differences comparing pre- and post-assessments were detected in the control group for both CMJ (ES [95%CI] = 0.04 [-0.30; 0.22], *P* = 0.74) and sprint performance (ES [95%CI] = 0.14 [-0.11; 0.39], *P* = 0.28). A group x time interaction was noted for CMJ (ES [95%CI] = 0.64 [0.28; 0.99], *P* = 0.001), whereas no significant differences were observed for sprint performance (ES [95%CI] = 0.02 [-0.32; 0.36], *P* = 0.85).

## DISCUSSION

This study examined the effects of a resistance training program on the physical performance of under-20 soccer players. With the exception of sprint speed, all conventional performance indicators (i.e., RS, RPF, and CMJ height) improved after the training intervention. In contrast, importantly, the SDef increased, which means that athletes became less prone to exert higher levels of relative force against submaximal loads.

We implemented a strength-power oriented scheme, starting with heavier loads/lower velocities (strength-phase) and finishing with lighter loads/higher velocities (power-phase). In line with previous studies [8, 13], this traditional approach was effective to enhance strength-related performance in soccer players, in both absolute and relative terms. Conversely, at the end of the training period, the SDef increased significantly in the experimental group, indicating that the difference between the force produced at the 1RM and at lighter loads (i.e., 40%1RM) increased in a greater proportion. From an applied perspective, this can be considered a training paradox, since the majority of soccer-specific actions (e.g., acceleration efforts or directional changes) are performed without any additional load, involving only the body-weight as resistance [6, 7]. Therefore, it would be preferred that SDef decreased or at least remained constant after a given training period. Anyway, improvements in maximum strength are positive, since a given absolute load would represent a lighter relative load and, hence, according to the load-velocity relationship, that load will be lifted at a higher velocity (i.e., the absolute loadvelocity relationship will be shifted to right and up) [1, 10]. However, an increased SDef also indicates that athletes are applying lower relative forces against lower %1RM and, thus, they will lift these relative loads at slower velocities (i.e., the relative load-velocity relationship will be shifted to left and down). It is possible that longer periods of a power-oriented phase are needed to improve the ability to apply greater levels of force against lighter loads [1, 4, 8]. Future studies are required to test this possibility or even to examine if increases in SDef are inevitable, being directly related to strength increases.

The improvements in CMJ height are also in accordance with previous research and can be explained by the strong relationships already found between strength and jump capacities [14, 15]. Likewise, the absence of positive changes in linear speed is supported by the literature when considering the negative effects of concurrent training on speed-related qualities, which is commonplace in soccer studies [16–18]. Nevertheless, for the first time, we raise the hypothesis that increases in SDef may compromise the players’ capability to use their strength potential at very-high velocities, which is critical to achieve superior sprint performances [19, 20]. As such, practitioners are advised to include this strength-derived measurement in their testing routines, as well as to develop training strategies aimed at simultaneously improving RS and reducing SDef. We recognize that, currently, there is no evidence that this affects sport performance or is even possible within the constraints of actual training practices. Furthermore, we acknowledge that the lack of more rigorous control of the total training load (i.e., total training and match time) may have affected our results. However, it is important to emphasize that this is a common limitation in training studies involving top-level athletes, especially soccer players.

In conclusion, maximum strength and SDef tend to increase concomitantly after a resistance training program. Stronger athletes are not necessarily able to use greater percentages of their peak-force against relatively lighter loads. This study is limited by several factors, including the lack of another experimental group to compare the effects of different training methods on SDef. Nonetheless, the inclusion of a control group allows us to verify that the SDef remains stable during a given training phase, when any type of structured resistance training program is applied. These novel findings open new avenues for research and evidence-based practices.

## CONCLUSIONS

A traditional strength-power oriented training program resulted in significant improvements in various strength-derived parameters and CMJ ability but, paradoxically, increased the SDef. Hence, athletes did not necessarily become more able (i.e., use greater percentages of peak-force) to apply force against relatively lighter loads. This occurrence may hamper the proper development of speed-related qualities or, at least, of the relative load-velocity relationship. Resistance exercises using very-light loads (e.g., 20–30% 1RM or only the body-mass as resistance) and few repetitions per set, moved as quickly as possible, may be required to reduce (or even maintain stable) the SDef after a structured training program. It is recommended that coaches regularly incorporate these exercises in their training routines.

## Conflict of interest

The authors have no conflict of interest to declare.
